# Supplementary material for: Contextual factors influencing a training intervention aimed at improved maternal and newborn healthcare in a health zone of the Democratic Republic of Congo
Source: PLoS One. 2021 Nov 29;16(11):e0260153. doi: 10.1371/journal.pone.0260153 (PMC8629278; doi:10.1371/journal.pone.0260153)
Supplement: S1 Appendix — (DOCX) [file pone.0260153.s001.docx]

# Interview guide

## Contextual factors influencing this training intervention

1. Please talk about the usefulness of the three-pillar training programme.

- Please give an example.
- Do you have another example?

1. Please talk about what facilitators there are, and how these influenced the implementation of the three-pillar training programme. Please consider both internal (at your own health facility) and external (outside your health facility) factors.

- Please give an example.
- Do you have another example?

1. Please talk about what barriers there are, and how these influenced the implementation of the three-pillar training programme. Please consider both internal and external factors.

- Please give an example.
- Do you have another example?

1. In your opinion, what is needed to enable the implementation of the three-pillar training programme?

- Please give an example.
- Do you have another example?
- What guidelines do you have?

1. Is there anything else you would like to add?
