# Supplementary material for: Contextual factors influencing a training intervention aimed at improved maternal and newborn healthcare in a health zone of the Democratic Republic of Congo
Source: PLoS One. 2021 Nov 29;16(11):e0260153. doi: 10.1371/journal.pone.0260153 (PMC8629278; doi:10.1371/journal.pone.0260153)
Supplement: Attachment — Submitted filename: Rebuttal letter.docx [file pone.0260153.s002.docx]

**Resubmission of Manuscript Number: PONE-D-21-15167**

*Contextual factors influencing a training intervention aimed at improved maternal and newborn healthcare in a health zone of the Democratic Republic of Congo*

Dear Editor-in-Chief,

We appreciate the thoughtful comments by the two reviewers, and thank them each for the time dedicated to reading and providing feedback on our manuscript. Please find our explanations and edits in response to each of their suggestions. All edits are highlighted in yellow in the manuscript.

| **Reviewer #1** | **Response** |
| --- | --- |
| This paper reports on a training programme in MNH in DRC. It is an interesting read but in itself there is nothing new about evaluating the impact of a training programme, as we know that teaching/training works. The authors instead have looked at incentives for training. | Thank you for your comment. This paper is about the context in which a training intervention takes place, and not about evaluating the impact of the training intervention itself. The impact of the training intervention will be reported on in another paper. |
| MMR figures are given for 2014 but there is data available from WHO for 2017, could this be used instead | There might be more recent data, but we prefer to use the attest data that the government of DRC still uses. |
| The methods section is sound and clearly written | Thank you very much. |
| In the results section 3 generic categories are given and the tables provided give clarity to how these were arrived at. Category one relates to financial incentives being effective, despite what the authors say that this is relatively new evidence, it has been reported by WHO as being effective as far back as 2010. | Thank you for your observation. We agree, we have removed the word “unexpected” and replaced it with “main”. (Discussion Paragraph 4).  Concerning “relatively new evidence”: Please note that we do not state that it is new evidence, but that the Cochrane review was recently published (2019). |
| There could be more discussion on the relative merits of financial incentives versus professional incentives (that is knowledge acquisition for doing a better job). This would bring in the 3rd pillar of the training more (strengthen the healthcare professionals’ self-reflection skills and self-confidence). | The aim of this study was to explore contextual factors influencing a training intervention consisting of 3 pillars of which the 3^rd^ was reflection in groups based on a process-oriented group reflection model were the participants reflect on self-selected care situation. It is not within these reflections that the concerns of financial incentives was stated, it is in our analysis of the focus group discussion data.  We have clarified about Pillar 3, see Method, Intervention, last sentence. |
| There needs to be more theoretical discussion around why women access improved services (for example issues of trust) | This is a very important issue, which we have elaborated on in a new paragraph in the discussion, see discussion paragraph 7. |
| There is nothing really new in the idea that this type of training programme needs government and local managerial support. | Yes, we agree, but in this specific DRC context where the training programme was conducted, it has not to our knowledge been discussed among the local authorities. |
| The authors mention the need for adequate physical space, electricity etc but then in the results mention this as part of service delivery and not just training needs. This needs to be a separate category which could emphasise more the difficulty of implementing training without an enabling environment. | Thank you very much for bringing this to our attention. We noticed that we had reported on the effect of the training programme, which is not the aim of this contextual study. We have removed inadequate text and re written; see subcategory Inadequate physical space and electricity, first paragraph. |
| The article does seem to concentrate on financial rather than non financial incentives and I wonder to make it stand out more if the real emphasis should be on non financial. This may yield a more original discussion and also link into to why if 80% of women in DRC do have a hospital assisted birth that the MMR ratio is still so high. Perhaps this relates to professional attitude in practice and that could be interesting to read. it may also be due to poor facilities and equipment and some discussion of this would be good. | Thank you for this interesting comment. This study focuses on contextual factors that influence a training programme. Your comment that this relates to professional attitude, which we agree to. However, this is relevant to discuss in our coming planned paper, which will focus on the effects of the training programme. |
| **Reviewer #2** | **Response** |
| it is a well written manuscript. the subject matter is relevant and important the importance of contextual characteristics required to design and execute a successful training for a major impacts well highlighted in a era where significant resources are being channeled to competency training in an effort to improve QOC ,this manuscript is very useful | Thank you very much! |
| **OTHER** | |
|  | Some other minor corrections have been made and they are yellow marked. |
